# Developing doctors: what are the attitudes and perceptions of year 1 and 2 medical students towards a new integrated formative objective structured clinical examination?

**DOI:** 10.1186/s12909-016-0542-3

**Published:** 2016-01-28

**Authors:** Daniel S. Furmedge, Laura-Jane Smith, Alison Sturrock

**Affiliations:** University College London Medical School, 74 Huntley Street, London, WC1E 6AU UK

**Keywords:** Assessment, OSCE, Undergraduate, Qualitative, Professionalism, Feedback

## Abstract

**Background:**

Objective Structured Clinical Examination (OSCE) is a core component of undergraduate medical student assessment. With increased emphasis on integrated programmes, more courses include OSCEs in the traditionally ‘preclinical’ years. The acceptability and impact of such assessment methods at this stage of training is unknown.

**Methods:**

In 2013 and 2014 University College London Medical School piloted a formative, integrated OSCE in years 1 and 2 of the undergraduate medical degree programme. This study with a sequential exploratory design aimed to explore the acceptability and impact of such an OSCE in the early years of medical school. 1280 students completed the OSCE and were invited to complete a questionnaire exploring their views of the OSCE (response rate 96.6 %). Four focus groups, each with five or six participants (22 in total) were subsequently held to further explore themes. Data was independently transcribed and coded using thematic analysis.

**Results:**

Students were strongly in favour of the introduction of an OSCE in addition to existing assessments. Six overarching themes were identified: application of knowledge and skills; OSCE as an experience; OSCE as a process; a learning curve; becoming a doctor; and creating an effective OSCE.

**Conclusions:**

Results strongly support the role of OSCE early in the medical course with many benefits reported. An OSCE at this stage aligns with the vision of integrated medical education which includes early patient contact and introduction of clinical and professional skills. It also fosters feelings of responsibility and professional identity amongst students.

**Electronic supplementary material:**

The online version of this article (doi:10.1186/s12909-016-0542-3) contains supplementary material, which is available to authorized users.

## Background

Objective structured clinical examination (OSCE) has become a mainstay of assessment in medical and healthcare education reflecting a competency-based paradigm [1]. A useful tool, OSCE has been described as the ‘gold standard’ for assessment of clinical competence [2, 3]. Many of the benefits of OSCEs are related to their feasibility, flexibility and adaptability. They are used in different specialities, to test different skills and domains across various settings and can follow either a formative or summative approach [4].

In the United Kingdom the majority of students are undergraduates and broadly spend the first two years learning preclinical basic sciences before moving to ‘clinical school’. Traditionally OSCE has been used in the later predominantly ‘clinical’ years of medical school. Some medical schools have introduced OSCE earlier in line with the increasing integration of medical education and the move away from a ‘Flexnerian’ separation between the preclinical and clinical components of the medical course [5–7]. Despite this movement, there is a distinct paucity of published research relating to the use of the OSCE in this unique period of early training.

There are several studies in the literature which relate specifically to ‘preclinical’ OSCE in healthcare and medical education though these often relate to a description of the OSCE, or confirming the reliability, validity or feasibility of the OSCE at this stage [4, 8–11]. There are studies which explore student perceptions towards OSCE, but these focus on OSCE in the clinical years. These studies found that although stressful, OSCE was highly acceptable to students, was better received than many other examination types, tested clinical skills, and allowed students to identify weaknesses [12–16]. There is a predilection for the reporting of organisation or examination specific details such as station length or content, rather than generalisable features in these studies.

University College London Medical School (UCLMS) identified that Bachelor of Medicine, Bachelor of Surgery (MBBS) year one and two students had no practical assessment of clinical skills despite the presence of increasing volumes of practical and communication skills, professionalism and other clinical elements in the year one and two curriculum. The first and sole formative OSCE appeared in the first clinical year and was peer-led (an OSCE designed and led by final year medical students with faculty supervision delivered at the end of a two week ‘orientation to clinical medicine’ module). This omission demonstrated a lack of constructive alignment within the course and it was noted, unsurprisingly that students were placing emphasis on learning those elements of the curriculum in the early years which were formally assessed. With strong evidence in support of OSCE as a tool, along with the changing practice of other medical schools, a decision was made to introduce OSCE into year one and two of the undergraduate curriculum. The challenge in writing OSCE stations was to integrate assessment of relevant knowledge with a clinical context.

This study focuses on the perceptions of year one and two students about the OSCE, in order to explore the acceptability and educational impact of such a novel integrated OSCE in the early years of medical school. The results and discussion focus on this generalisable aspect rather than specific institution or examination feedback which is being used at a local level for quality improvement purposes.

## Methods

This was a mixed methods study with a sequential exploratory design consisting of questionnaires with five-level Likert and free text responses and focus groups.

A new, formative OSCE was introduced to both year one and two of the undergraduate medicine course two months before summative examinations. The OSCE in each year consisted of eight active five-minute stations, in order to have a throughput of up to 350 students on one single day, in double circuits, across three sites. A full circuit was 40 min duration. Stations were developed by a working group of academics and clinicians and were designed to sample multiple areas of knowledge, skills and behaviours using a curriculum blueprint. There was a predefined focus on integration of basic and clinical sciences and its formative nature meant that its primary aim was to aid student learning and not contribute to grades or outcomes in any way. Students received their individual scores and further oral feedback was given to the whole year group by faculty.

In the exploration of the experiences of students in sitting the formative OSCE, we adopted a constructivist epistemology. This posits that students generate knowledge and meaning from interactions between their experiences and ideas [17]. We chose this as we were particularly interested in student’s experiences of a new form of assessment, and how they made sense of this within their journey towards formulating a professional identity. Within this framework we undertook a thematic analysis. The analysis was inductive, such that data rather than theory drove development of the coding framework. We followed an explanatory sequential design, such that initial survey research immediately after the formative assessment provided data which was then elaborated by later in-depth qualitative focus groups [18].

The questionnaire was developed by faculty following a literature review on early years OSCEs and assessment of scientific principles through methods other than written assessment. This review highlighted concerns in the medical education community about the feasibility of integrating basic science into an OSCE format, and about acceptability to students of an OSCE early in their student career. This informed the development of the questionnaire, which was split into two sections: general feedback and station/examination specific. Questionnaires were informally piloted using faculty to assess readability and usability but were not formally validated as the primary aim of this questionnaire was to inform local course evaluation.

1280 students sat either the year 1 or 2 OSCE over two years, 2013 and 2014. Immediately following the OSCE, before they left the examination centre, all students were invited to complete a paper-based questionnaire about their experiences. This created four cohorts of questionnaire response (year 1 2013, year 2 2013, year 1 2014, year 2 2014). Year 1 students from 2013 repeated the questionnaire after their second OSCE in year 2 in 2014 and this has been taken into account in the statistical analysis. Questionnaires were employed at this stage rather than focus groups for logistical reasons to minimise non-response bias. Access to all students at the point of exiting the examination ensured that a wide breadth of opinion was obtained and free-text responses were encouraged at this point to allow for elaboration and further detail. Questionnaires comprised statements with Likert scales measuring levels of agreement on a five level scale from strongly disagree to strongly agree. Questions related to the overall experience of the OSCE with specific questions about acceptability and usefulness of the exercise. Further free-text boxes were included to seek more detail about the experience. 1236/1280 students completed questionnaires, a response rate of 96.6 %.

Mean and median averages with standard deviations were calculated for all pooled Likert statement responses (Table 1). A Spearman’s correlation and subsequent factor analysis using the Varimax rotation were calculated for all student responses to Likert scale items; this was done to assess whether the questions all assessed an underlying factor, or whether there was a more complicated factor structure. This was important as this was a novel questionnaire which had not been validated. A Mann–Whitney test to examine for potential differences between the 2013 year 1 cohort and the 2014 year 2 cohort (the majority of whom were the same students).

**Table 1:** Student responses to Likert scale questionnaire items

	Strongly disagree	Disagree	Neutral	Agree	Strongly agree	n	Median	Mean	Standard deviation
	1	2	3	4	5				
My level of anxiety before this exam was detrimental to my performance	102 (8.3 %)	362 (29.3 %)	393 (31.8 %)	290 (23.5 %)	87 (7.1 %)	1234	3	2.92	1.07
The exam tested my progress	20 (2 %)	91 (7.4 %)	276 (22.4 %)	652 (52.9 %)	194 (15.7 %)	1233	4	3.74	0.87
The exam was a worthwhile exercise	15 (1.2 %)	27 (2.2 %)	144 (11.7 %)	534 (43.3 %)	512 (41.6 %)	1232	4	4.22	0.82
The exam gave me a good chance to demonstrate my knowledge/ability	12 (0.9 %)	94 (7.6 %)	314 (25.5 %)	612 (49.6 %)	201 (16.3 %)	1233	4	3.73	0.86
Thid type of exam is enjoyable	114 (9.2 %)	241 (19.5 %)	379 (30.7 %)	367 (29.7 %)	133 (10.8 %)	1234	3	3.13	1.13
This type of exam is appropriate for this stage of training	22 (1.8 %)	95 (7.7 %)	343 (27.8 %)	575 (46.7 %)	197 (16 %)	1232	4	3.67	0.9
This exam was acceptable to me	19 (1.5 %)	90 (7.3 %)	304 (24.7 %)	580 (47 %)	240 (19.5 %)	1233	4	3.75	0.9
The exam balanced integration of clinical skills with basic science	4 (0.3 %)	41 (3.3 %)	186 (15 %)	611 (49.6 %)	391 (31.7 %)	1233	4	4.09	0.79

Initial free-text results from 2013 questionnaires were transcribed manually and analysed in a line-by-line analysis separately by two researchers who identified initial themes. These researchers then met to discuss and agree themes, refining these and together devising an initial coding framework. Codes were manually assigned pending a final coding framework being agreed upon after focus group data was available.

Four subsequent focus groups of five or six participants each (22 students in total) were held to both triangulate data and to further explore prominent themes in more detail. Focus groups occurred six weeks after the OSCE and questionnaire administration for two reasons: to see if views differed after a time period had elapsed and to allow students to have received and reflected on their results. A semi-structured interview schedule (see Additional file 1) was devised based on themes generated from the questionnaire free text response analysis. Focus groups were divided by year to facilitate free discussion of year-specific information that could be used for evaluative and improvement purposes. All students who had participated in the OSCE were invited to attend a focus group via e-mail sent out by the course administrator. A £10 voucher was offered for participating. Students consented to participate in focus groups and were allocated to groups on a first-come-first-served basis.

Focus groups were conducted in university tutorial rooms by two clinical teaching fellows (junior clinicians working within the medical school) all with experience of qualitative interviewing; no further specific training was given for these focus groups. Focus groups were recorded using a Dictaphone and recordings were transcribed by an independent professional stenographer within three weeks of the focus groups occurring. Field notes were not formally taken. Two researchers reviewed themes initially generated from the free text sections of the survey research, against the new data from the first focus group, and fitted new themes into a revised coding structure. They discussed and agreed the final coding framework which was then applied to the whole dataset using QSR International’s NVivo10 qualitative analysis Software [19]. Data from both free-text responses and focus groups was then independently coded by two researchers and a medical student. Analysis of all free text responses and focus group transcripts continued until data saturation, such that no new themes were generated from analysis of further data.

This study was a service evaluation and did not therefore require formal ethics committee approval in accordance with UCL regulations. Accordingly it was not submitted formally to an ethics committee.

## Results

### Quantitative data

Data is available on 305 year 1 students in 2013, 322 year 2 students in 2014 (largely the same students), 304 year 1 students in 2014 and 303 year 2 students in 2013. Student responses to questionnaire items are found in Table 1. Totals for each column do not always equal 1236 due to a small number of unanswered or ‘left blank’ items. The strongest agreement were with the statements ‘the exam was a worthwhile exercise’ (84.9 % agreed or strongly agreed), ‘the exam balanced integration of clinical skills with basic science’ (81.3 % agreed or strongly agreed). The weakest positive statement was ‘this type of exam is enjoyable (40.5 % agreed or strongly agreed).

There are eight variables. These are strongly correlated suggesting that students valued the OSCE as an experience (Table 2).

**Table 2:** Spearman’s correlation of student responses to Likert scale questionnaire items

Spearman’s correlation	Worthwhile	Demonstrate	Appropriate	Acceptable	Integration	Anxiety	Enjoyable
Test progress	0.41**	0.45**	0.33**	0.36**	0.38**	−0.08*	0.24**
Worthwhile		0.49**	0.43**	0.52**	0.43**	−0.08*	0.28**
Demonstrate			0.40**	0.43**	0.38**	−0.15**	0.42**
Appropriate				0.54**	0.38**	−0.14**	0.39**
Acceptable					0.52**	−0.15**	0.34**
Integration						−0.11**	0.24**
Anxiety							−0.30**

**: *p* < 0.001

*: *p* < 0.01

Factor analysis was carried out on all the responses using Varimax rotation. (Table 3).

**Table 3:** Factor analysis on all student responses to Likert scale questionnaire items using Varimax rotation

Item	Loading on factor 1^a^	Loading on factor 2^a^
Test progress	0.69	
Worthwhile	0.78	
Demonstrate	0.69	
Appropriate	0.64	
Acceptable	0.75	
Integration	0.70	
Anxiety		−0.87
Enjoyable	0.36	0.70

^a^Weights less than 0.3 not shown

This suggests there are two factors: a hedonic response (loading on anxiety and enjoyment) and an overall rating of the exam (all other items). Two new scores were thus created by averaging over these factors (reverse coding anxiety).

Median scores (with interquartile ranges) are given for each cohort in Tables 4 and 5. These suggest that both year 1 and 2 students ‘liked’ the OSCE with the year 1 students ‘liking’ it slightly more than those in year 2.

**Table 4:** Median Likert score 1–5 (with interquartile ranges) for each cohort

Cohort	Test progress	Worthwhile	Demonstrate	Appropriate	Acceptable	Integration	Anxiety	Enjoyable
year 1 students in 2013	4 (3–4)	4 (4–5)	4 (3–4)	4 (3–4)	4 (3–4)	4 (4–5)	3(2–4)	3 (2–4)
year 1 students in 2014	4 (3–4)	4 (4–5)	4 (3–4)	4 (3–4)	4 (3–4)	4 (4–5)	3(2–4)	3 (2–4)
year 2 students in 2013	4 (3–4)	4 (4–5)	4 (3–4)	4 (3–4)	4 (3–4)	4 (4–5)	3(2–4)	3 (2–4)
year 2 students in 2014	4 (3–4)	4 (4–5)	4 (3–4)	4 (3–4)	4 (3–4)	4 (4–5)	3(2–4)	3 (2–4)

**Table 5:** Pooled overall ratings with hedonic response

Cohort	Overall rating	Hedonic
year 1 students in 2013	4 (3.5-4.3)	3 (2.5-3.5)
year 1 students in 2014	4 (3.5-4.3)	3 (2.5-3.5)
year 2 students in 2013	3.8 (3.5-4.3)	3 (2.5-4)
year 2 students in 2014	3.8 (3.5-4.2)	3 (2.5-4)

Comparing the 2013 year 1 scores with the 2014 year 2 scores, *i.e.* the same cohort of students (Table 6) suggested that there was no effect on results for the 2014 year 2 cohort on having done an OSCE the previous year. The only difference between these groups was that by year 2 there was a lower average score on “The exam gave me a good chance to demonstrate my knowledge/ability”.

**Table 6:** Mann–Whitney test comparing 2013 year 1 score with 2014 year 2 scores

Item	*z*	*P*
Test progress	1.9	0.057
Worthwhile	1.7	0.093
Demonstrate	2.9	0.004
Appropriate	1.3	0.2
Acceptable	1.9	0.059
Integration	1.8	0.072
Anxiety	0.3	0.8
Enjoyable	1.6	0.11
*Overall rating*	1.9	0.052
*Hedonic*	1.0	0.3

### Qualitative data

Free text from all of the 1236 questionnaire responses and data from four focus groups, each with five or six participants (22 in total) were analysed. First year students in 2013 became second year students in 2014 and therefore completed the questionnaire twice; after their year 1 OSCE in 2013 and their year 2 OSCE in 2014. Six overarching themes running throughout the data were identified: OSCE as an experience, OSCE as a process, application of knowledge and skills, learning curve, becoming a doctor, and creating an effective OSCE. These will be explored in more depth below.

### OSCE as an experience

Students described the OSCE as fun, enjoyable and satisfying. Whilst acknowledging that it was a challenging and stressful experience, they felt it was worthwhile and realistic.

*I think it’s actually quite nice to like, sometimes be challenged on things that you don’t know. It’s quite good practice to just see how well you deal when you don’t know what’s going on.*

(**Year 2 focus group 1**)

They enjoyed the ‘break from the books’ offered by the OSCE and the opportunity to experience a formative OSCE in a low stakes environment, significantly reducing anxiety about future OSCE experiences.

### OSCE as a process

The OSCE was seen by students to be a patient centred process and they were positive about the focus of the exam on patient care, which they perceived to be less obvious in written assessments.

*…like your patient doctor interaction which is the most important. After all you know NHS is patient centred medicine, so I mean it’s right that to have an OSCE tests such skills*

(**Year 1 focus group 2**)

Students noted awkwardness in interacting with examiners they knew but generally found examiners friendly and sympathetic. Although the exam was felt to be fair overall, they believed that, despite best efforts to standardise the exam, there was some variability across sites and between examiners.

### Application of knowledge and skills

Students overwhelmingly reported the OSCE to be a place where they could apply integrated knowledge, ‘showing how rather than just knowing how’ and demonstrate their skills and understanding.

*…you felt more like you were able to demonstrate a bit more the breadth of your knowledge*

(**Year 2 focus group 2**)

Even if they couldn’t answer specific questions within a station or hadn’t performed well in written examinations, they felt they were able to show what they did know. For some, there was a realisation that although not strong performers in written assessments, the OSCE provided an area to flourish, justifying their place as a student doctor.

*…I never really tend to do that well in exams because like… …I don’t know, it just never happens. But I’m a lot more able to explain things to people, so that kind of made me feel more confident that I actually do know my stuff*

(**Year 1 focus group 1**)

The OSCE was seen as a place where understanding, not recall was tested and acknowledged as the place where a variety of skills and knowledge was tested which could not be assessed elsewhere.

### Learning curve

The OSCE enabled students to highlight weaknesses and gaps in their knowledge before summative examinations. Students consistently reported consolidating skills not tested elsewhere, and subsequently realised the value of practising skills.

*… it’s better to find out now, what your weaknesses are now and be able to work on how you introduce yourself, just like basic things like that rather than to get into year four and realise ‘Oh my God, I don’t even know how to introduce myself!’*

(**Year 1 focus group 1**)

They reported new study and revision techniques which centred on group work, communication, interaction, teamwork and peer-learning.

*I think it encouraged quite a lot more group work and working in teams, I think that’s really positive*

(**Year 1 focus group 1**)

Students felt they developed skills in thinking on their feet in challenging situations and likened this to the real life scenarios they would face as a doctor. They described feeling more confident and able to cope in unknown situations.

*It made you think on your feet which is an experience otherwise not provided by other forms of assessment*

(**Year 1 questionnaire**)

There was renewed interest in, and greater perceived relevance of, professional skills teaching strands such as communication skills, ethics and law and clinical skills. Previously not tested, these were now seen in the context of becoming a doctor.

### Becoming a doctor

Becoming a doctor was a strong theme throughout. Students highlighted on numerous occasions that the experience of sitting the OSCE made them feel like doctors. Their learning, now contextualised and relevant, actually meant something and was being put into practice.

*…but also just in terms of becoming a good doctor, what is clinically relevant and what will come up over and over again in our careers*

(**Year 2 focus group 2**)

*I liked performing the more practical stations and in a way it made me feel like I was becoming a doctor. I liked talking to people I hadn’t met before as if they were patients as this is going to be something I do in the future*

(**Year 1 questionnaire**)

Students described the realisation that medicine is about more than knowledge, and began to talk about the privilege and complexities of being in a position of authority, having to care for patients, and being accountable.

*If a patient comes to you, you should be able to explain how a drug acts and you should be able to look at a pathology slide and interpret it. Because at the end of the day if you don’t know how to then how are you going to explain to patients what the results are?*

(**Year 1 focus group 1**)

#### Creating an effective OSCE

This theme related directly to what students felt was needed to improve the OSCE. More information, more preparation and practice, more feedback and more stations were common suggestions.

## Discussion

### Summary of results

Overall students found the OSCE a worthwhile, acceptable and rewarding experience which they stated reduced anxiety about future summative OSCEs. They enjoyed applying and integrating knowledge in what they described as challenging and realistic situations. The OSCE stimulated and directed learning and fostered positive feelings about becoming a doctor, a responsibility to patients and professionalism. It gave a renewed relevance to integrated professional teaching strands, previously overlooked by students until later in the course.

Students highlighted some negatives about the OSCE experience which tended to relate to local aspects of the examination such as specific station content or the need for more preparatory information. The overall impression was strongly in favour of continuing OSCE at this stage and these feelings persisted at six weeks and at one year.

Students scored the OSCE favourably in the questionnaire, valuing the experience as a whole. Overall year one students favoured the OSCE slightly more than the year two students and this was similar for the year group which repeated the process in both years. Possible explanations include the impact of more experience in medical school and in assessment, a slightly more jaded outlook and perhaps, exam fatigue. The fact that demonstration of knowledge or ability also scored lower amongst the second year students may relate to the different content of the exams in year one and two. This has been the focus of some internal development work.

### OSCE as a process and a learning experience

Many of the themes identified mirror those seen in other qualitative studies concerning OSCE, particularly its utility in assessment, its ability to assess clinical skills and its value in identifying weaknesses [10, 12, 16]. This study has affirmed that these benefits are applicable across years and with varied levels of experience. This study also reinforced previous studies which found that OSCE is perceived as educational. The placement of this OSCE two months before the summative OSCE provided an opportunity to address gaps in knowledge and skills.

Students reported that the OSCE was a whole new way of thinking, and learning. Their description of adopting new strategies for learning, despite this being a formative assessment, were consistent with previous literature, in that they used social networks to learn, rather than just books [20]. Social networks and friendship have been found to be an important influencer on learning and attainment; earlier development of these networks may therefore have a beneficial effect and is a key area for further research [21].

Resilience is a hot topic in contemporary medical education and has been identified as a key area in which medical education institutions should direct focus [22]. Although this is a vast area with multiple components including identity formation and wellbeing, the exposure of students to a high-stress examination in a relatively low stakes environment at this early stage was described favourably by students. Previously, students have reported being ‘thrown in at the deep end’ when first entering clinical studies [23] and so their descriptions of how it allowed them to test personal ability to cope and adapt in a stressful environments, and to develop skills in thinking on their feet, are encouraging. Early patient exposure has been reported to reduce the shock and stress of entering a clinical environment and improve non-analytical reasoning [24–26]. Further research about how early OSCE exposure could contribute to development of non-analytical reasoning, and the development of resilience is indicated.

### Becoming a doctor

To students, in spite of increasing curricular integration, the ‘preclinical’ years can often seem far removed from what they came to medical school to do: become a doctor. Strong themes which emerged from this data included students’ feelings about ‘becoming a doctor’ and early realisation of their responsibilities and a duty of care towards patients. This is extremely encouraging; with professional identity formation in medicine a dynamic process influenced by multiple factors [27], the potential role of the OSCE in contributing to the development of proto-professionals is important [28].

The skills tested in the OSCE are universally and more easily aligned to the General Medical Council Good Medical Practice framework than written assessments. This guidance is the overriding set of professional standards to which all UK doctors must adhere [29]. With accountability and fitness to practise regulations now affecting all medical students, it is vital that this code of conduct and its application pervades the whole of medical training, allowing students to develop and adopt these behaviours from the outset. Aligning assessment methods with these aims is an effective way to introduce such strands of professionalism earlier.

### Early OSCE-isation

Medical students in the preclinical years rapidly adapt to being medical students, learning the new norms of being in this exclusive group. They become a homogenous body, adopting certain behaviours and actions via multiple processes and influences. Such homogenisation clearly has both advantages and disadvantages [30, 31]. One significant disadvantage is the dehumanisation of doctors with subsequent treatment of patients as objects rather than human beings. It is difficult to know how OSCE influences this process. On one hand, an early focus on patients, immersing students into near real-life situations might promote the relevance of their learning in a patient-centred way as seems to be the case from this study. On the other, promoting OSCE-focused behaviour with standardised interactions may have the opposite effect.

Students reported interactions with simulated ‘patients’ and scenarios as realistic. This is despite these students’ limited exposure to a real clinical environment. They also reported that a key benefit was the reduction in anxiety and experience in sitting an OSCE before later high-stakes examinations. This early exposure to OSCE, whilst allaying anxiety may be problematic and suggests student focus on assessment. To sit an OSCE is to perform and the over-use of OSCE has been highlighted as an area for caution [32]. Students develop a standardised OSCE technique and may adopt a pseudo-empathic approach which may spill over into real practice [33]. Although this concept is not investigated further here, and in spite of the perceived benefits in this study, further research into the effects of such early OSCE exposure in students with limited real patient contact is essential.

There were a number of limitations of this study. This was a single-centre study evaluating one specific OSCE at one large medical school which limits generalisability of our findings. However, the OSCE was designed using principles recognised by the medical education community and through adapting examples from other institutions. In addition, the high response rates of the questionnaire mean that we have captured a wide range of views. One group of students completed the OSCE in both years, as first year students in 2013 and as second year students in 2014. This means they completed the questionnaires twice and due to the anonymous nature of the data collection cannot be individually identified. This has been acknowledged within the statistical analysis. The nature of qualitative data analysis is subjective and leads to possible bias in interpretation and synthesis of comments, indeed those researchers who facilitated and analysed the focus groups were also involved in the running of the OSCE itself which must be noted. Attempts to reduce this bias included the use of a third coder, a final year medical student who had no previous experience of or involvement in this OSCE.

## Conclusion

The incorporation of formative OSCEs into the traditionally preclinical early years of medical school is highly acceptable to medical students and seems to have a positive educational impact. In particular the OSCE has a positive effect on student learning and enables them to contextualise and assimilate their integrated knowledge into the complex process of becoming a doctor. It also fosters feelings of professional identity, responsibility and a duty of care to patients. Medical schools are encouraged to ensure they have an OSCE in the preclinical phase of their course which aims to integrate scientific knowledge with clinical skills.

## Additional file

Additional file 1:
**Focus group schedule.** (RTF 39 kb)

## Abbreviations

MBBS: Bachelor of medicine, bachelor of surgery
OSCE: Observed structured clinical examination
UCL: University College London
UCLMS: University College London Medical School
