# Supplementary material for: Developing doctors: what are the attitudes and perceptions of year 1 and 2 medical students towards a new integrated formative objective structured clinical examination?
Source: BMC Med Educ. 2016 Jan 28;16:32. doi: 10.1186/s12909-016-0542-3 (PMC4730723; doi:10.1186/s12909-016-0542-3)
Supplement: Additional file 1: — Focus group schedule. (RTF 39 kb) [file 12909_2016_542_MOESM1_ESM.rtf]

Year 1 & 2 OSCE Focus Group Schedule
1. How did you find the experience of an OSCE overall?
2. What was particularly positive about the OSCE?
3. What was particularly negative about the OSCE?
4. Was the exam fair?
5. How has the OSCE changed your learning styles, if at all?
6. Will your approach to study change in your next year of the MBBS programme as a result of your experiences and if so how?
7. What did you think of the balance of stations and the integration between science and clinical skills? Did it work?
8. What would need to be done before the OSCE became a summative exam,? i.e. an exam where the marks count towards you passing the year and your overall mark
9. Apart from the obvious answer of giving out markschemes and details of exact stations which we are unable to do, what further information would help?
10. Did you go to any OSCE preparation sessions run by MEDSOC or anyone else? If so why, if not why not? If so, did they help?
11. How useful was the feedback lecture to the year?
12. Knowing that we cannot give you a breakdown of marks, how could feedback be improved?
